# Serum 25-Hydroxyvitamin D_3_ and D_2_ and Non-Clinical Psychotic Experiences in Childhood

**DOI:** 10.1371/journal.pone.0041575

**Published:** 2012-07-25

**Authors:** Anna-Maija Tolppanen, Adrian Sayers, William D. Fraser, Glyn Lewis, Stanley Zammit, John McGrath, Debbie A. Lawlor

**Affiliations:** 1 MRC Centre for Causal Analyses in Translational Epidemiology, School of Social and Community Medicine, University of Bristol, Bristol, United Kingdom; 2 School of Social and Community Medicine, University of Bristol, Bristol, United Kingdom; 3 Norwich Medical School, University of East Anglia, Norwich, United Kingdom; 4 MRC Centre for Neuropsychiatric Genetics and Genomics, Cardiff University, Cardiff, United Kingdom; 5 Queensland Centre for Mental Health Research and Department of Psychiatry and Queensland Brain Institute, University of Queensland, St Lucia, Australia; Chiba University Center for Forensic Mental Health, Japan

## Abstract

**Objective:**

Non-clinical psychotic experiences are common and distressing. It has been hypothesized that early life vitamin D deficiency may be a risk factor for psychosis-related outcomes, but it is not known if circulating concentrations of 25-hydroxyvitamin D (25(OH)D) during childhood are associated with psychosis-related outcomes or whether the two different forms of 25(OH)D, (25(OH)D_3_ and 25(OH)D_2_, have similar associations with psychosis-related outcomes.

**Methods:**

We investigated the association between serum 25(OH)D_3_ and 25(OH)D_2_ concentrations and psychotic experiences in a prospective birth cohort study. Serum 25(OH)D_3_ and 25(OH)D_2_ concentrations were measured at mean age 9.8 years and psychotic experiences assessed at mean age 12.8 years by a psychologist (N = 3182).

**Results:**

Higher 25(OH)D_3_ concentrations were associated with lower risk of definite psychotic experiences (adjusted odds ratio: OR (95% confidence interval: CI) 0.85 (0.75–0.95)). Higher concentrations of 25(OH)D_2_ were associated with higher risk of suspected and definite psychotic experiences (adjusted odds ratio: OR (95% confidence interval: CI) 1.26 (1.11, 1.43)). Higher 25(OD)D_2_ concentrations were also weakly associated with definite psychotic experiences (adjusted OR (95% CI) 1.17 (0.96, 1.43), though with wide confidence intervals including the null value.

**Conclusions:**

Our findings of an inverse association of 25(OH)D_3_ with definite psychotic experiences is consistent with the hypothesis that vitamin D may protect against psychosis-related outcomes.

## Introduction

Non-clinical psychotic experiences occur in approximately 10% of the population and people experiencing such symptoms may be at higher risk of clinical psychotic disorders later in life. [1]–[3] If a substantial proportion of individuals in non-clinical samples have psychotic experiences occurring as a result of pathological abnormalities underlying the etiology of schizophrenia, we might expect shared risk factors for both phenotypes. [4] Low vitamin D status during early life has been suggested to be a risk factor for schizophrenia. [5] Previous studies have assessed associations between serum 25-hydroxyvitamin D (25(OH)D) measured from neonatal blood samples [6] or vitamin D supplementation during first year of life [7] and later risk of schizophrenia. However, it is unknown if serum 25(OH)D concentrations in childhood are associated with psychotic experiences. The only previous study that we are aware of, examining the association of vitamin D and psychotic experiences, was a cross-sectional study of adults, examining the association of dietary intake of vitamin D. [8] Serum 25(OH)D concentrations are mainly determined by ultraviolet B (UVB) exposure and therefore the possibility that individuals with psychotic experiences were less likely to eat diets rich in vitamin D (rather than the dietary vitamin D affecting psychotic experiences) cannot be excluded.

25(OH)D consists of (a) 25(OH)D_3_ (synthesised from vitamin D_3_ mainly obtained from synthesis in skin in response to UVB exposure, but also from supplements and some food sources such as oily fish) and (b) 25(OH)D_2_ (synthesised from vitamin D_2_ which is obtained from plant food sources and supplements). It is valuable to examine the associations of both forms of 25(OH)D with outcomes, because supplements of either vitamin D_2_ or D_3_ are readily available and if it is found that circulating concentration of one form is more strongly associated with outcomes than the other, and this is subsequently determined to be causal, then future trials of effectiveness of supplementation should use the one with the likely stronger effect. To our knowledge previous studies have not examined the association between 25(OH)D_2_ concentrations and cognitive outcomes or whether 25(OH)D_3_ and 25(OH)D_2_ differ from each other in their associations with psychotic experiences. The aims of this study were to investigate the prospective associations of 25(OH)D_3_ and 25(OH)D_2_ with non-clinical psychotic experiences and to compare the magnitudes of association of the two forms of 25(OH)D with each other. Because vitamin D, together with parathyroid hormone (PTH) regulates calcium and phosphate homeostasis [9], [10] we investigated whether PTH, calcium and phosphate were associated with psychotic experiences and whether the associations of 25(OH)D_3_ or 25(OH)D_2_ were independent of PTH, calcium or phosphate concentrations.

## Methods

### Population

The Avon Longitudinal Study of Parents and Children (ALSPAC) is a population-based birth cohort from South West England. The cohort consisted of 14062 live births from 14541 enrolled pregnant women who were expected to give birth between April 1, 1991, and December 31, 1992. [11] From age 7, all children were invited for an annual assessment of physical and psychological development. Parents gave informed consent and ethical approval was obtained from the ALSPAC Law and Ethics Research Committee and the National Health Service local research ethics committee.

**Figure 1:**
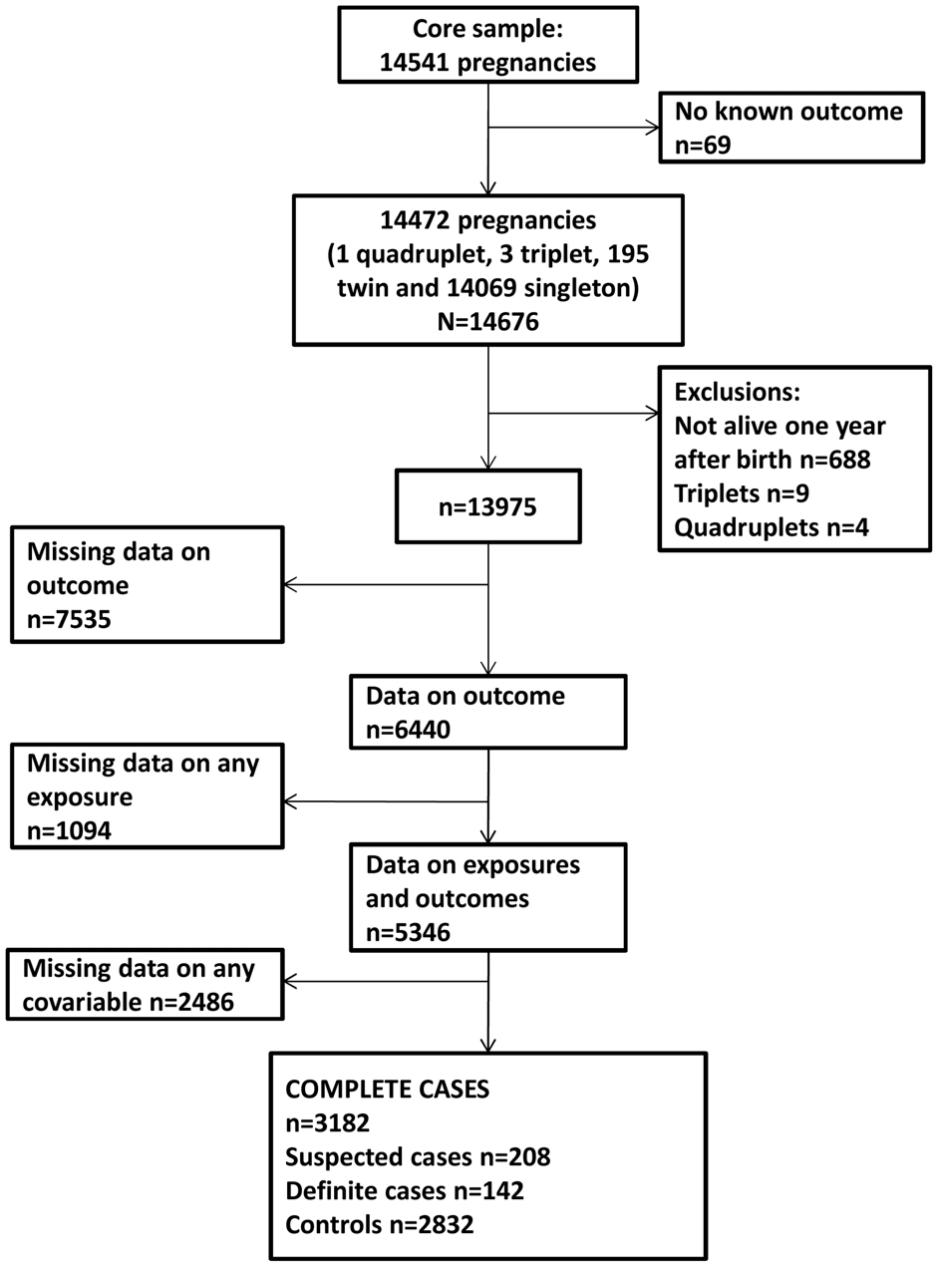
Flow of participants.

Single and twin births were included in this study; the very small number of triplets and quadruplets were not included for reasons of confidentiality. Figure 1 shows the flow of participants through the cohort follow-up and numbers available for analyses. In this study we included 3182 participants with complete data on exposures, outcome and co-variables.

### Exposures and Blood Based co-variables

Serum 25(OH)D_3_, 25(OH)D_2_, PTH, phosphate and calcium were assayed on non-fasting blood samples collected at mean age 9.9 years for the majority of participants. If no samples were available from the 9.9 years assessment, samples from mean age 11.8 years or, secondly, the 7.6 years assessment, were used. The mean age at sample collection in the whole study sample was 9.8 years (standard deviation: SD 1.1). After the phlebotomy, the samples were immediately frozen at −80°C in freezers temperature control and electronic temperature monitoring. Assays were performed in 2010 after a maximum of 12 years in storage at −80°C with no previous freeze-thaw cycles. 25(OH)D_2_, 25(OH)D_3_ and deuterated internal standard were extracted from serum samples after protein precipitation using zinc sulphate with Isolute C18 solid phase extraction cartridges (Biotage AB, Uppsala, Sweden). Potential interfering compounds were removed by initial elution with 50% methanol followed by elution of the vitamins using 10% tetrahydrofuran in acetonitrile. Dried extracts were reconstituted prior to injection into a high performance liquid chromatography tandem mass spectrometer including Waters 2777 autosampler (Waters Acquity, Manchester UK) in conjunction with a Knauer Smartline 1000 quaternary pump and 6300 Sample preparation unit (Knauer, Berlin, Germany), fitted with Knauer 10 µm C18 guard column Waters Sunfire 3.5 µm 2.1 mm×50 mm C18 column and Micromass Quattro Ultima Platinum (KRSS, Manchester, UK) fitted with a Z Spray electrospray ionisation inlet. The following mass to charge ratios in the multiple reaction mode were used: 413.2>395.3, 401.1>383.3 and, 407.5>107.2 for 25(OH)D_2_, 25(OH)D_3_, and deuterated internal standard, respectively. Inter-assay coefficients of variation (CV) for the assay were <10% across a working range of 1–250 ng/mL for both 25(OH)D_3_ and 25(OH)D_2_. The sensitivities of the assays were defined by estimating the signal to noise ratio obtained at low 25(OH)D_2_ and 25(OH)D_3_ concentrations. Measurements were performed in the Bioanalytical Facility of Royal Liverpool University Hospital laboratory which meets the performance target set by the Vitamin D External Quality Assessment Scheme (DEQAS) Advisory Panel for 25(OH)D assays.

Total serum calcium, phosphate and albumin concentrations were measured by standard laboratory methods on Roche Modular analysers (Roche Diagnostics Ltd, West Sussex, UK). Serum calcium was adjusted for albumin using a normogram of calcium and albumin distributions of the samples analysed in the clinical chemistry laboratory where the measurements were performed. Intact parathyroid hormone (iPTH(1–84)) was measured by electrochemiluminescent immunoassay on an Elecsys 2010 immunoanalyzer (Roche, Lewes, UK). Inter-assay CV was <6% from 2–50 pmol/L. The assay sensitivity (22% CV for duplicates) was 1 pmol/L.

### Outcomes

Psychotic experiences were assessed using the semi-structured psychosis-like symptoms (PLIKS) interview administered by trained psychologists at mean (SD) age of 12.8 (0.2) years as described previously. [12] Semi-structured interviews are most closely approximated to clinical assessments of psychotic phenomena in clinical psychiatry as they allow for cross-examination of individuals to ensure that the experiences described are psychotic phenomena, unlike self-report assessments and structured interviews that over-estimate the occurrence of such experiences. [13], [14] The PLIKS interview asks about the occurrence of visual and auditory hallucinations, delusions and experiences of thought interference during the previous 6-months. Twelve core items were probed with stem questions derived from the Diagnostic Interview Schedule for Children version IV [15] and the Schedules for Clinical Assessment in Neuropsychiatry version 2.0. [16] These were modified slightly after piloting [further details available at Academic Unit of Psychiatry, University of Bristol (http://www.bristol.ac.uk/psychiatry/staff/zammit/documents/pliks.pdf)]. Interviewers rated symptoms as not present/suspected/definitely present. Symptoms were included only if they were not attributable to effects of sleep or fever and were rated as definite only when a credible example was provided. The average kappa value for interrater reliability within our study was 0.72. [14].

### Confounding Factors

We considered gender, age, ethnicity (white/non-white), socioeconomic position, family history of depression or schizophrenia, exposure to UVB, body mass index (BMI), cognitive function and puberty stage to be potential confounders because of their known associations with 25(OH)D concentrations and psychotic experiences. Thus, we defined confounders as common causes of exposure and outcome and based the selection on existing literature, as suggested by Hernán *et al,* [17] instead of using any arbitrary statistical criteria. Data on head of household social class, ethnicity, maternal and paternal education and family history of depression, schizophrenia or other psychiatric problem were obtained from parent-completed questionnaires. Data on time spent outdoors and protection from solar UVB exposure (use of sunblock, covering clothing or hat and avoidance of midday sun) was obtained from parent-completed questionnaires at mean age of 8.5 years. BMI was calculated from height and weight measured at the same time as blood samples were taken. Total IQ score in Wechsler Intelligence Scale for Children (WISC-III UK version) was assessed at mean age 8.5. Puberty stage was assessed by parental report using Tanner staging [18] of breast development and pubic hair on repeat occasions. We used data from the questionnaire closest to the time of phlebotomy.

### Statistical Analysis

Statistical analyses were conducted with Stata 11.0 (Stata Corp LP, College Station, TX USA).

**Figure 2:**
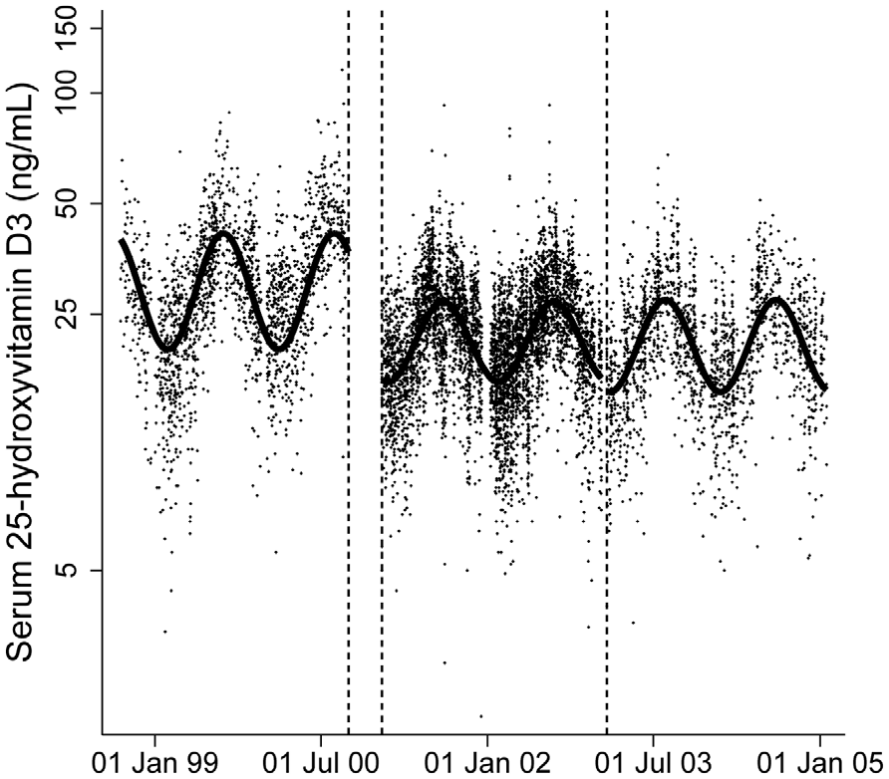
Seasonal variation in serum 25(OH)D_3_ concentrations. Dots represent individual observations and the average seasonal value is indicated by the line.

25(OH)D_3_ concentrations followed sinusoidal seasonal variation with peaks during summer and troughs during winter (Figure 2). Therefore 25(OH)D_3_ was modeled according to date of blood sampling using linear regression with trigonometric sine and cosine functions. 25(OH)D_3_ was log_e_ transformed to reduce heteroscedasticity. The residual was then used as 25(OH)D_3_ exposure variable in regression analyses. To include all participants on whom 25(OH)D_2_ was assayed, those with a value below the detectable limit of the assay (0.5 ng/mL) were given a value of 0.5 ng/mL and indicated using a binary covariable in all regression models (n = 1129, 35.5%). For the main analyses we compared associations of seasonal adjusted 25(OH)D_3_ with psychotic experiences to those of 25(OH)D_2_ and also present associations of total 25(OH)D with outcomes for better comparability across studies. In supplementary analyses we also report associations unadjusted (for season) 25(OH)D_3_ with psychotic experiences. This examines whether adjustment for season change explains any of the association. In order to take account of age differences at the time of assessment we generated age- and gender-standardised standard deviation scores for serum 25(OH)D_3_, 25(OH)D_2,_ calcium, phosphate and PTH using the internal cohort data with age in one month categories.

The associations of potential confounders with age- and gender-standardised 25(OH)D_3_, 25(OH)D_2_, calcium, phosphate and PTH concentrations was assessed with linear regression and the associations of confounders with psychotic experiences with logistic regression. To examine the main associations of interest of exposures with outcome and to compare associations of different exposures we performed a non-parametric bootstrap procedure in conjunction with multivariable logistic regression, based on 1000 replications. The bootstrapping procedure enabled us to statistically compare associations of 25(OH)D_3_ with psychotic experiences to those of 25(OH)D_2_ with psychotic experiences. The difference between the association of 25(OH)D_3_ and 25(OH)D_2_ was calculated from the bootstrap replicate distribution. Beta estimates and standard errors were empirically calculated from the mean and standard deviation of the bootstrap distribution, respectively. All *P* values were calculated using bootstrap means and standard errors and compared to a z-distribution.

We examined two outcomes: a combined outcome of both suspected or definite psychotic experiences versus none, and definite psychotic experiences versus none (i.e. in the latter those with suspected psychotic experiences were removed from the analyses). To numerically compare the associations of two forms of 25(OH)D, beta coefficients from the logistic regression models were multiplied by log_e_(2). The results are interpreted as the odds ratio of psychotic experiences (suspected or definite versus none or definite versus none) per doubling of exposure. Linearity of associations was tested with a likelihood ratio test comparing a model with quintiles of standardized exposure as indicator variables to model with exposure quintiles as a continuous score.

## Results

The median (interquartile range) for unadjusted 25(OH)D_3_, seasonal adjusted 25(OH)D_3_, 25(OH)D_2_ and total 25(OH)D in the analysis sample (N = 3182) were 22.2 (17.4–27.5), 24.9 (24.7–25.1), 1.3 (0.5–2.7) and 24.1 (19.4–29.4) ng/mL, respectively. The median (interquartile range) for serum phosphate, albumin-adjusted calcium and PTH were 1.53 (1.43–1.64) mmol/L, 2.37 (2.31–2.45) mmol/L and 4.5 (3.4–5.8) pmol/L, respectively. Distribution of 25(OH)D_2_ concentrations was positively skewed, while 25(OH)D_3_ concentration was approximately normally distributed; as noted above both were log transformed for the analyses. The proportion of participants with total 25(OH)D concentration below 20 ng/mL was similar among all 7560 participants with serum measurements (n = 2162, 28.6%) and among the 3182 participants included in this study (n = 896, 28.2%). In the analysis cohort of 3182 participants, 350 (11.0%) had the combined outcome of suspected or definite psychotic experiences and 142 (4.8%) had definite psychotic experiences; these proportions were similar to those in the total 6440 participants who provided data on psychotic experiences (some of whom were excluded because of missing exposure or covariable data): 742 (11.5%) had suspected or definite psychotic experiences and 300 (4.7%) had definite psychotic experiences.

Table S1 shows the univariable associations of potential confounders with 25(OH)D_2_ and 25(OH)D_3_ and the associations of potential confounders with PTH, calcium and phosphate are shown in Table S2. Higher BMI and non-white ethnicity were associated with lower 25(OH)D_2_ concentrations and higher PTH concentrations. White children had higher 25(OH)D_3_ and lower PTH concentrations. Children from higher socioeconomic backgrounds had higher 25(OH)D_3_ and lower 25(OH)D_2_ and calcium concentrations. Those who avoided midday sun more often had lower concentrations of 25(OH)D_3_ and calcium and higher concentrations of phosphate and 25(OH)D_2_. Children who spent less time outdoors in the summer had lower 25(OH)D_3_ concentrations.

Table S3 shows the univariable associations of potential confounders with psychotic experiences. Children with higher IQ and those with white ethnicity were less likely to have experienced definite psychotic experiences. Lower parental education and parental history of mental health problems were associated with higher risk of suspected/definite and definite psychotic experiences. Children who spent more time outdoors during summer weekends were slightly more likely to have suspected/definite and definite psychotic experiences.

Table 1 shows the multivariable associations between exposures and psychotic experiences. Serum 25(OH)D_3_ concentrations were inversely associated with definite psychotic experiences in analyses that controlled for age, gender, season and ethnicity (Model 1). The association remained similar after adjusting for outdoor exposure and BMI (Model 2), socioeconomic position, family history of mental health problems and IQ (Model 3) and other analytes including 25(OH)D_2_ (Model 4). This association appeared linear across the entire distribution of 25(OH)D_3_ (*P* for evidence of non-linearity  = 0.64). When the outcome was extended to include both suspected as well as definite psychotic experiences there was no clear association of 25(OH)D_3_ with this outcome.

**Table 1:** Prospective association of seasonal adjusted 25(OH)D_3_, 25(OH)D_2_, phosphate, calcium and PTH concentrations (assessed at mean age 9.9 years) with psychotic experiences (PLIKS) (assessed at age 12.8 years).

Exposure	Outcome	Odds ratio of outcome per doubling of exposure (95%CI)			
		Model 1	Model 2	Model 3	Model 4
25(OH)D_3_	Suspected/definite PLIKS	0.95 (0.88, 1.02)	0.94 (0.87, 1.01)	0.95 (0.88, 1.03)	0.96 (0.88, 1.03)
	Definite PLIKS	0.86 (0.77, 0.95)	0.85 (0.75, 0.95)	0.85 (0.76, 0.95)	0.85 (0.75, 0.95)
25(OH)D_2_	Suspected/definite PLIKS	1.27 (1.13, 1.43)	1.27 (1.11, 1.44)	1.26 (1.12, 1.43)	1.26 (1.11, 1.43)
	Definite PLIKS	1.21 (1.00, 1.48)	1.21 (0.98, 1.47)	1.19 (0.98, 1.43)	1.17 (0.96, 1.43)
Total 25(OH)D	Suspected/definite PLIKS	0.95 (0.89, 1.03)	0.95 (0.88, 1.02)	0.95 (0.88, 1.02)	0.95 (0.88, 1.03)
	Definite PLIKS	0.90 (0.82, 1.00)	0.89 (0.81, 0.98)	0.89 (0.80, 0.99)	0.89 (0.80, 0.99)
Albumin-adjusted calcium	Suspected/definite PLIKS	1.04 (0.95, 1.12)	1.04 (0.96, 1.13)	1.03 (0.95, 1.12)	1.01 (0.93, 1.09)
	Definite PLIKS	1.05 (0.92, 1.19)	1.05 (0.92, 1.18)	1.05 (0.92, 1.19)	1.05 (0.91, 1.21)
Phosphate	Suspected/definite PLIKS	1.05 (0.97, 1.13)	1.05 (0.98, 1.13)	1.06 (0.98, 1.15)	1.05 (0.96, 1.14)
	Definite PLIKS	0.98 (0.89, 1.09)	0.99 (0.89, 1.10)	0.99 (0.88, 1.12)	0.97 (0.87, 1.09)
Parathyroid hormone	Suspected/definite PLIKS	1.02 (0.94, 1.11)	1.02 (0.94, 1.11)	1.02 (0.93, 1.11)	1.00 (0.91, 1.10)
	Definite PLIKS	1.02 (0.91, 1.13)	1.03 (0.92, 1.15)	1.03 (0.92, 1.15)	1.01 (0.89, 1.15)

N = 3182 (208 cases with suspected/definite PLIKS) and N = 2974 (142 cases with definite PLIKS; in these analyses suspected cases are removed from the analyses).

Model 1 is unadjusted (all exposures are standardised for age and gender and 25(OH)D_3_ is adjusted for season and ethnicity).

Model 2 as Model 1 plus adjustment for ethnicity, time spent outdoors during summer (age 8.5 years), use of sunblock, hat, covering clothing, avoidance of midday sun and BMI.

Model 3 as Model 2 plus adjustment for head of household social class, mother’s and partner’s education, family history of mental health problems and puberty stage.

Model 4 as Model 3 plus adjustment for serum concentrations of other hormones/metabolites which are related to vitamin D homeostasis (i.e. each of the exposures listed in the first column is mutually adjusted for all others in the first column).

Higher serum 25(OH)D_2_ concentrations were positively associated with definite psychotic experiences in all models, but the estimates were imprecise and 95% confidence intervals included the null value. When the outcomes were extended to include both suspected and definite cases there was a statistically robust association in all models, which appeared linear across the distribution of 25(OH)D_2_ (*P* for evidence of a non-linear association  = 0.18).

There was strong statistical evidence that the inverse association of 25(OH)D_3_ with definite psychotic experiences differed from the positive association of 25(OH)D_2_ association with this outcome (*P = *0.008). There was also evidence that the associations of 25(OH)D_3_ and 25(OH)D_2_ with suspected/definite psychotic experiences statistically differed from each other (*P* = 0.005).

Associations of total 25 (OH) D with outcomes were broadly similar to those seen with 25(OH)D_3_ (Table 1). None of PTH, calcium or phosphate concentrations were associated with psychotic experiences (Table 1) and hence there was no evidence that these mediated any of the observed associations of total 25(OH)D, 25(OH)D_3_ or 25(OH)D_2_ with outcomes (Model 4 in Table 1).

The associations of unadjusted (for season) 25(OH)D_3_ (Table 2) were broadly similar to those of seasonal adjusted 25(OH)D_3_.

**Table 2:** Prospective association of unadjusted (for season) 25(OH)D_3_ concentrations (assessed at mean age 9.9 years) with psychotic experiences (PLIKS) (assessed at age 12.8 years). N = 3182 (208 cases with suspected/definite PLIKS) and N = 2974 (142 cases with definite PLIKS; in these analyses suspected cases are removed from the analyses).

Exposure	Outcome	Odds ratio of outcome per doubling of exposure (95%CI)			
		Model 1	Model 2	Model 3	Model 4
25(OH)D_3_	Suspected/definite PLIKS	0.88 (0.79, 0.97)	0.87 (0.78, 0.96)	0.87 (0.77, 0.96)	0.88 (0.79, 0.98)
	Definite PLIKS	0.88 (0.79, 0.97)	0.86 (0.78, 0.95)	0.87 (0.78, 0.95)	0.88 (0.78, 0.98)

Model 1 is unadjusted (25(OH)D_3_ is adjusted for age and gender).

Model 2 as Model 1 plus adjustment for ethnicity, time spent outdoors during summer (age 8.5 years), use of sunblock, hat, covering clothing, avoidance of midday sun and BMI.

Model 3 as Model 2 plus adjustment for head of household social class, mother’s and partner’s education, family history of mental health problems and puberty stage.

Model 4 as Model 3 plus adjustment for 25(OH)D_2,_ phosphate, albumin-adjusted calcium and parathyroid hormone.

## Discussion

Consistent with the hypothesis that better vitamin D status, indicated by higher concentrations of 25(OH)D, protects against schizophrenia we found an inverse association of serum concentrations of 25(OH)D_3_ and total 25(OH)D with definite psychotic experiences in childhood, though the association was weaker when suspected cases were also included in the outcome. Contrary to expectations from previous studies of total 25(OH)D or those of dietary or supplement intake of vitamin D with schizophrenia, we found that higher concentrations of 25(OH)D_2_ were associated with psychotic experiences. However, it should be noted that the results are presented per doubling of exposure and thus the associations of 25(OH)D_3_ and 25(OH)D_2_ (15% risk decrease and 26%risk increase in the adjusted model, respectively) were modest.

The associations were robust to adjustment for a range of potential confounding factors that we selected based on appropriate methods. [17] Decisions regarding such selection can be difficult, e.g. with 25(OH)D_3_ adjustment for seasonality and UVB exposure might be considered as overadjustment, given their role in determining 25(OH)D_3_. However, the associations were similar when we used 25(OH)D_3_ without adjustment for seasonality (Table S2) and also when we removed indicators of UVB exposure (data not presented). Adjustment for seasonality in studies assessing the association of 25(OH)D with outcomes can improve statistical efficiency and reduce confounding when the outcome is also likely to be seasonally patterned and does not lead to overadjustment. [19] There has been debate about whether BMI or adiposity influences 25(OH)D concentrations or the other way around (i.e. 25(OH)D influences adiposity. A recent Mendelian randomization study supports the direction being form greater adiposity causing lower 25(OH)D, [20] than the other way round, which supports inclusion of BMI as a potential confounder.

To our knowledge one previous study has examined the association of vitamin D with psychotic experiences. [8] In that large cross-sectional study of women, dietary vitamin D intake, estimated with food frequency questionnaires, was inversely associated with psychotic experiences. It is difficult to directly compare these results with ours as we find the inverse association only with 25(OH)D_3_, which is largely determined by UVB, rather than diet. Another prospective study found that maternal report of vitamin D supplementation in the first year of life was protective against schizophrenia in adulthood in men but not in women. [7] Since the exposure in that cohort study was maternal report of vitamin D_3_ and/or D_2_ supplement use with no detailed data on which supplement the children received, [21] it is impossible to know whether that represented predominantly an association of vitamin D_3_ or D_2_ with outcome. Notably, in these previous studies the outcome was assessed in adulthood, [7], [8] while we studied the association with psychotic experiences in childhood/early adolescence.

We had the opportunity to explore the relative associations of both 25(OH)D_3_ and 25(OH)D_2_ with psychotic experiences. This is important because on the basis of associations with bone outcomes [22], [23] and affinity for vitamin D binding protein [23], [24] it has been suggested that vitamin D_3_ is more potent than vitamin D_2_ but nutritional supplements are available in both forms and for non-bone outcomes any differences between the associations of two forms of 25(OH)D are, as yet, unknown. We have previously shown that although 25(OH)D_3_ was the major contributor to total 25(OH)D in this cohort, 25(OH)D_2_ makes an important contribution to total 25(OH)D concentrations in some children, especially those with low total 25(OH)D concentrations. [25].

Our data linking outdoor behaviour with 25(OH)D_3_ is consistent with the hypothesis that the children in this cohort largely received their vitamin D_3_ via sunlight exposure on the skin, not dietary intake. While over a third of the cohort had no detectable 25(OH)D_2,_ it was of interest to note that those with higher concentrations of this form of circulating 25(OH)D (which is exclusively derived from dietary sources such as fortified margarine and cereals as well as supplements) were more likely to report psychotic experiences. This finding may be a chance occurrence, given the lack of any previous studies with which to compare the results. It could reflect residual confounding by one or more characteristics that are related to increased dietary intake of vitamin D_2_ and also increased risk of psychotic experiences. It could be causal, but as yet we are unaware of any biological mechanism that would explain it or different associations of 25(OH)D_3_ and 25(OH)D_2_. We emphasise that this finding should be treated with caution unless replicated in other studies.

Experimental studies suggest that vitamin D is important for neuronal function and brain development [26]–[29]. The majority of the animal studies that have examined effects on neuronal function have been based on vitamin D receptor knock-out models or developmental vitamin D deficiency. The relevance of these studies to our findings depends on the extent to which the consequences of early life vitamin D deficiency in animal models, including lateral ventricle size/structure, increased cellular proliferation, reduced apoptosis, and altered neurogenesis [26]–[29] continue to operate in children and adolescents. There is some emerging evidence to suggest that low vitamin D status may impact on brain outcomes differently in the post-natal brain (e.g. vitamin D may be ‘neuroprotective’ and help the post-natal brain cope with stressors). [30], [31].

In interpreting our findings it is important to consider what psychotic experiences reflect. If they represent an early expression of pathological neurodevelopmental processes that later lead to schizophrenia, our results would have potential implications for predication and for understanding aetiology of schizophrenia. This possibility is supported by suggestions that people with non-clinical psychotic experiences represent a valuable and valid group for studying the aetiology of clinical psychosis, and the similarity of risk factors for non-clinical and clinical psychotic symptoms. [4] However, it is also true that in many individuals who report psychotic experiences, these phenomena are transient, and despite the clear association between earlier psychotic experiences and later clinical psychotic disorders, the majority of those with psychotic experiences do not progress to clinical disorders. [2], [3], [32], [33] Even if such experiences are not a useful indicator of future clinical outcomes, they are associated with distress and functional impairment, [34], [35] and may represent an important public health concern, in the same way that symptoms of depression and anxiety that do not reach thresholds used to formally diagnose depression do. [36] Thus, understanding more about the aetiology of such experiences is likely to be important.

The study has several important limitations. We lacked detailed information on supplement intake for the cohort members and thus were not able to infer how much of the circulatory 25(OH)D_3_ was derived from dietary sources. Although we were able to adjust for confounders, including measures of socioeconomic position and outdoor exposure, it is possible that these dimensions were not fully captured and there is some residual confounding. Reverse causality is possible, for example if those with psychotic experiences were less likely to go outdoors and be exposed to UVB as a result of these symptoms. However, the prospective nature of our study reduces this possibility and prospectively we actually found a weak positive association of reported time spent outdoors with psychotic experiences, making this possibility unlikely. Our findings may be due to chance and thus further replication in large prospective cohorts would be valuable, as would comparing the associations of both forms of 25(OH)D with hard endpoint of clinically diagnosed schizophrenia, in order to establish the true association with this disorder. Mendelian randomization studies, in which genetic variants that are robustly associated with circulating concentrations of 25(OH)D_3_ and 25(OH)D_2_ are used as instrumental variables to assess their causal effects on outcomes [37]–[39] would also be valuable, but these would require very large sample sizes. Ultimately, large randomised trials would be required to determine whether supplementation with vitamin D was an effective means of preventing psychosis-related outcomes. [40] Our findings do suggest that vitamin D_3_ would be the more appropriate supplement to be assessed in trials examining the effect of this vitamin on psychotic experiences if these further studies were to suggest causal protective effects of this form of vitamin D.

We only used data from a single measurement of exposures which may be inadequate, [41] although a single measurement may be a useful biomarker of season-specific vitamin D status over a longer time. [42]. Our sample included mainly white children (the number of non-white children inthe main analyses was 84 (2.6%)), which may limit the generalisability of the results, but does mean that residual confounding due to ethnicity is unlikely. Similar to other prospective cohort studies there was loss to follow-up and those who have attended follow-up clinics tend to be from higher socioeconomic groups. [11] However, the prevalence of psychotic experiences was similar in complete cases and those who were excluded due to missing data.

### Conclusions

In conclusion, our findings provide some support for an inverse association of total 25(OH)D and 25(OH)D_3_ concentrations with psychotic experiences, which if psychotic experiences are related to development of schizophrenia, also support a possible protective association of higher 25(OH)D_3_ concentrations with schizophrenia. The positive association of 25(OH)D_2_ concentrations with psychotic experiences was unexpected and is currently unexplained. Further replication of our findings in large prospective studies would be valuable, as would the use of studies, such as Mendelian randomized controlled trials and randomised controlled trials, that are more able to establish whether associations are likely to be causal or not.

## Acknowledgments

We are extremely grateful to all the families who took part in this study, the midwives for their help in recruiting them, and the whole ALSPAC team, which includes interviewers, computer and laboratory technicians, clerical workers, research scientists, volunteers, managers, receptionists and nurses.

## Supporting Information

Table S1 **Univariable associations between potential confounders and age and gender standardised serum 25-hydroxyvitamin D_3_ and D_2_ concentrations (N = 3182).**
(DOCX)

Table S2 **Univariable associations between potential confounders and age and gender standardised serum phosphate, calcium and PTH concentrations.**
(DOCX)

Table S3 **Univariable associations between potential confounders and suspected/definite (N = 3182) and definite (N = 2974)psychotic experiences (PLIKS).**
(DOCX)
