# Supplementary material for: Serum 25-Hydroxyvitamin D3 and D2 and Non-Clinical Psychotic Experiences in Childhood
Source: PLoS One. 2012 Jul 25;7(7):e41575. doi: 10.1371/journal.pone.0041575 (PMC3405076; doi:10.1371/journal.pone.0041575)
Supplement: Table S1 — Univariable associations between potential confounders and age and gender standardised serum 25-hydroxyvitamin D3 and D2 concentrations (N = 3182). (DOCX) [file pone.0041575.s001.docx]

**Table S1.** Univariable associations between potential confounders and age and gender standardised serum 25-hydroxyvitamin D_3_ and D_2_ concentrations (N=3182)

|  | Season-adjusted 25(OH)D_3_ | | 25(OH)D_2_ | |
| --- | --- | --- | --- | --- |
|  | SD change per SD/category change (95%CI) | *P* | SD change per SD/category change (95%CI) | *P* |
| BMI (kg/m^2^) | -0.01 (-0.02 to 0.01) | 0.36 | -0.02 (-0.03 to -0.01) | 0.001 |
| WISC full IQ score at age 8.5 | -0.05 (-0.78 to 0.68) | 0.90 | -0.75 (-1.48 to -0.02) | 0.044 |
| Non-white ethnicity | -0.55 (-0.78 to -0.32) | <0.001 | -0.08 (-0.32 to 0.16) | 0.52 |
| Head of household social class | |  |  |  |
| i | 0.08 (0.00 to 0.16) | <0.001 | -0.08 (-0.17 to 0.00) | 0.001 |
| ii | 0.00 (-0.10 to 0.10) |  | 0.07 (-0.03 to 0.17) |  |
| iii non-manual | -0.06 (-0.18 to 0.05) |  | 0.09 (-0.02 to 0.21) |  |
| iii manual | -0.25 (-0.41 to -0.08) |  | 0.21 (0.05 to 0.38) |  |
| iv/v | -0.31 (-0.56 to -0.06) |  | 0.38 (0.13 to 0.64) |  |
| Paternal education |  |  |  |  |
| None/CSE | -0.05 (-0.16 to 0.05) | 0.57 | 0.12 (0.01 to 0.23) | 0.010 |
| Vocational | 0.10 (-0.08 to 0.28) |  | -0.12 (-0.31 to 0.06) |  |
| O level | 0.15 (0.01 to 0.28) |  | -0.06 (-0.18 to 0.05) |  |
| A level | 0.10 (-0.03 to 0.22) |  | -0.08 (-0.20 to 0.05) |  |
| Degree | 0.07 (-0.05 to 0.20) |  | -0.21 (-0.34 to -0.08) |  |
| Maternal education |  |  |  |  |
| None/CSE | 0.00 (-0.13 to 0.14) | 0.36 | 0.09 (-0.05 to 0.23) | 0.001 |
| Vocational | 0.05 (-0.15 to 0.25) |  | -0.02 (-0.22 to 0.18) |  |
| O level | 0.09 (-0.06 to 0.24) |  | -0.01 (-0.16 to 0.14) |  |
| A level | 0.00 (-0.15 to 0.15) |  | -0.16 (-0.32 to -0.01) |  |
| Degree | 0.00 (-0.15 to 0.16) |  | -0.16 (-0.32 to -0.01) |  |
| Child normally wears hat whilst out in the sun |  |  |  |  |
| Always | 0.03 (-0.06 to 0.12) | 0.07 | -0.06 (-0.15 to 0.03) | 0.22 |
| Usually | 0.06 (-0.05 to 0.16) |  | 0.07 (-0.04 to 0.18) |  |
| Sometimes | -0.02 (-0.13 to 0.09) |  | 0.06 (-0.05 to 0.17) |  |
| Never | -0.17 (-0.35 to 0.01) |  | 0.13 (-0.06 to 0.31) |  |
| Child normally wears something to cover the skin whilst out in the sun |  |  |  |  |
| Always | 0.00 (-0.10 to 0.10) | 0.39 | -0.02 (-0.12 to 0.08) | 0.62 |
| Usually | 0.04 (-0.07 to 0.15) |  | 0.04 (-0.08 to 0.15) |  |
| Sometimes/Never | 0.05 (-0.06 to 0.17) |  | -0.01 (-0.12 to 0.11) |  |
| Child normally uses sunblock whilst out in the sun |  |  |  |  |
| Always | 0.06 (0.01 to 0.11) | 0.10 | 0.01 (-0.04 to 0.06) | 0.99 |
| Usually | -0.05 (-0.13 to 0.03) |  | -0.06 (-0.14 to 0.02) |  |
| Sometimes/Never | -0.09 (-0.20 to 0.03) |  | 0.05 (-0.07 to 0.17) |  |
| Child normally avoids midday sun |  |  |  |  |
| Always | 0.03 (-0.06 to 0.13) | 0.08 | -0.03 (-0.13 to 0.07) | <0.001 |
| Usually | -0.05 (-0.16 to 0.07) |  | 0.06 (-0.06 to 0.17) |  |
| Sometimes | 0.05 (-0.06 to 0.16) |  | 0.01 (-0.11 to 0.12) |  |
| Time spent outdoors in summer during school weekdays >3h/day | 0.03 (-0.04 to 0.10) | 0.44 | 0.04 (-0.04 to 0.11) | 0.34 |
| Time spent outdoors in summer during weekends >3h/day | 0.18 (0.06 to 0.31) | 0.004 | 0.08 (-0.05 to 0.21) | 0.21 |
| Time spent outdoors in summer during holidays >3h/day | 0.23 (0.11 to 0.35) | <0.001 | 0.01 (-0.11 to 0.14) | 0.86 |
| Family history of depression/schizophrenia |  |  |  |  |
| None | -0.04 (-0.20 to 0.12) | 0.27 | 0.01 (-0.27 to 0.30) | 0.70 |
| Depression | -0.04 (-0.13 to 0.04) |  | 0.05 (-0.04 to 0.13) |  |
| Schizophrenia | -0.08 (-0.37 to 0.20) |  | -0.18 (-0.47 to 0.11) |  |
| Puberty stage at serum measurement |  |  |  |  |
| 1 | 0.06 (0.00 to 0.12) | 0.30 | 0.02 (-0.04 to 0.07) | 0.18 |
| 2 | -0.04 (-0.13 to 0.04) |  | -0.02 (-0.10 to 0.07) |  |
| 3 | -0.01 (-0.11 to 0.09) |  | -0.02 (-0.13 to 0.08) |  |
| 4-5 | -0.12 (-0.29 to 0.04) |  | -0.15 (-0.32 to 0.02) |  |
