# Supplementary material for: Serum 25-Hydroxyvitamin D3 and D2 and Non-Clinical Psychotic Experiences in Childhood
Source: PLoS One. 2012 Jul 25;7(7):e41575. doi: 10.1371/journal.pone.0041575 (PMC3405076; doi:10.1371/journal.pone.0041575)
Supplement: Table S2 — Univariable associations between potential confounders and age and gender standardised serum phosphate, calcium and PTH concentrations. (DOCX) [file pone.0041575.s002.docx]

**Supplementary Table 2.**Univariable associations between potential confounders and age and gender standardised serum phosphate, calcium and PTH concentrations

|  | | | Phosphate | | | Albumin-adjusted calcium | | | | | Parathyroid hormone | |
| --- | --- | --- | --- | --- | --- | --- | --- | --- | --- | --- | --- | --- |
|  | | | SD change per SD/category change (95%CI) | *P* | | SD change per SD/category change (95%CI) | | | *P* | | SD change per SD/category change (95%CI) | *P* |
| BMI (kg/m^2^) | | | 0.00  (-0.01 to 0.01) | 0.82 | | 0.00  (-0.01 to 0.01) | | | 0.81 | | 0.01  (0.00 to 0.02) | 0.02 |
| WISC full IQ score at 8.5 years | | | 0.00  (0.00 to 0.00) | 0.09 | | 0.00  (0.00 to 0.00) | | | 0.17 | | 0.00  (0.00 to 0.00) | 0.16 |
| Non-white ethnicity | | | -0.14  (-0.38 to 0.10) | 0.24 | | -0.09  (-0.33 to 0.15) | | | 0.46 | | 0.35  (0.11 to 0.59) | 0.004 |
| Head of household social class | | | |  | |  | | |  | |  |  |
| i | | | 0.03  (-0.06 to 0.11) | 0.09 | | -0.06  (-0.14 to 0.03) | | | 0.35 | | -0.03  (-0.11 to 0.06) | 0.19 |
| ii | | | 0.00  (-0.10 to 0.10) |  | | 0.07  (-0.03 to 0.17) | | |  | | 0.00  (-0.10 to 0.10) |  |
| iii non-manual | | | -0.05  (-0.17 to 0.06) |  | | 0.05  (-0.06 to 0.17) | | |  | | 0.01  (-0.11 to 0.12) |  |
| iii manual | | | -0.12  (-0.29 to 0.05) |  | | 0.03  (-0.14 to 0.19) | | |  | | 0.06  (-0.11 to 0.23) |  |
| iv/v | | | -0.12  (-0.37 to 0.14) |  | | 0.18  (-0.07 to 0.44) | | |  | | 0.21  (-0.04 to 0.47) |  |
| Paternal education | | | |  | |  | | |  | |  |  |
| None/CSE | | | 0.01  (-0.10 to 0.12) | 0.44 | | 0.12  (0.01 to 0.22) | | | 0.02 | | 0.03  (-0.08 to 0.14) | 0.72 |
| Vocational | | | -0.12  (-0.30 to 0.06) |  | | -0.15  (-0.33 to 0.03) | | |  | | -0.12  (-0.31 to 0.06) |  |
| O level | | | -0.02  (-0.16 to 0.12) |  | | -0.12  (-0.26 to 0.02) | | |  | | -0.06  (-0.19 to 0.08) |  |
| A level | | | 0.00  (-0.13 to 0.13) |  | | -0.10  (-0.23 to 0.02) | | |  | | 0.09  (-0.06 to 0.24) |  |
| Degree | | | 0.01  (-0.12 to 0.14) |  | | -0.19  (-0.32 to -0.06) | | |  | | -0.06  (-0.19 to 0.06) |  |
| Maternal education | | |  |  | |  | | |  | |  |  |
| None/CSE | | | -0.02  (-0.16 to 0.12) | 0.24 | | 0.06  (-0.08 to 0.19) | | | 0.02 | | 0.09  (-0.04 to 0.23) | 0.38 |
| Vocational | | | 0.08  (-0.12 to 0.28) |  | | -0.03  (-0.23 to 0.17) | | |  | | -0.28  (-0.48 to -0.08) |  |
| O level | | | -0.03  (-0.19 to 0.12) |  | | -0.02  (-0.17 to 0.13) | | |  | | -0.13  (-0.28 to 0.02) |  |
| A level | | | 0.03  (-0.12 to 0.19) |  | | -0.06  (-0.22 to 0.09) | | |  | | -0.13  (-0.28 to 0.03) |  |
| Degree | | | 0.07  (-0.09 to 0.23) |  | | -0.14  (-0.30 to 0.01) | | |  | | -0.05  (-0.20 to 0.11) |  |
| Child normally wears hat whilst out in the sun | | | | | |  | | |  | |  |  |
| Always | | | 0.06  (-0.03 to 0.15) | 0.28 | | 0.06  (-0.02 to 0.15) | | | 0.20 | | -0.09  (-0.18 to 0.00) | 0.03 |
| Usually | | | -0.08  (-0.19 to 0.03) |  | | -0.08  (-0.19 to 0.03) | | |  | | 0.07  (-0.04 to 0.18) |  |
| Sometimes | | | -0.04  (-0.15 to 0.07) |  | | -0.09  (-0.20 to 0.02) | | |  | | 0.09  (-0.02 to 0.19) |  |
| Never | | | -0.18  (-0.37 to 0.00) |  | | -0.06  (-0.25 to 0.12) | | |  | | 0.23  (0.05 to 0.42) |  |
|  |  |  | | |  | |  |  | |  |  |  |
| Always | | | -0.06  (-0.16 to 0.04) | 0.25 | | 0.00  (-0.10 to 0.10) | | | 0.57 | | -0.02  (-0.12 to 0.08) | 0.95 |
| Usually | | | 0.07  (-0.05 to 0.18) |  | | 0.01  (-0.10 to 0.13) | | |  | | 0.00  (-0.11 to 0.12) |  |
| Sometimes/Never | | | 0.08  (-0.04 to 0.19) |  | | -0.02  (-0.14 to 0.10) | | |  | | 0.01  (-0.11 to 0.12) |  |
| Child normally uses sunblock whilst out in the sun | | | | | |  | | |  | |  |  |
| Always | | | 0.0  1(-0.04 to 0.07) | 0.65 | | 0.00  (-0.05 to 0.05) | | | 0.88 | | -0.02  (-0.07 to 0.03) | 0.54 |
| Usually | | | -0.03  (-0.12 to 0.05) |  | | -0.02  (-0.10 to 0.07) | | |  | | -0.02  (-0.10 to 0.07) |  |
| Sometimes/Never | | | -0.01  (-0.13 to 0.11) |  | | 0.00  (-0.12 to 0.12) | | |  | | 0.06  (-0.06 to 0.18) |  |
| Child normally avoids midday sun | | | | | |  | | |  | |  |  |
| Always | | | -0.05  (-0.15 to 0.05) | <0.001 | | -0.02  (-0.11 to 0.08) | | | <0.001 | | -0.06  (-0.16 to 0.04) | 0.62 |
| Usually | | | 0.03  (-0.08 to 0.15) |  | | 0.01  (-0.11 to 0.12) | | |  | | 0.06  (-0.05 to 0.18) |  |
| Sometimes | | | 0.09  (-0.02 to 0.21) |  | | 0.02  (-0.10 to 0.13) | | |  | | 0.03  (-0.09 to 0.14) |  |
| Never | | | 0.01  (-0.16 to 0.18) |  | | 0.04  (-0.13 to 0.20) | | |  | | 0.07  (-0.09 to 0.24) |  |
| Time spent outdoors in summer during school weekdays >3h/day | | | 0.01  (-0.07 to 0.09) | 0.78 | | 0.05  (-0.03 to 0.12) | | | 0.23 | | -0.01  (-0.09 to 0.06) | 0.72 |
| Time spent outdoors in summer during weekends >3h/day | | | 0.03  (-0.10 to 0.16) | 0.61 | | 0.06  (-0.07 to 0.19) | | | 0.33 | | -0.10  (-0.23 to 0.03) | 0.13 |
| Time spent outdoors in summer during holidays >3h/day | | | 0.07  (-0.06 to 0.19) | 0.28 | | 0.00  (-0.12 to 0.13) | | | 0.93 | | -0.06  (-0.18 to 0.07) | 0.36 |
| Family history of depression/schizophrenia | | | | | |  | | |  | |  |  |
| None | | | -0.02  (-0.30 to 0.27) | 0.65 | | -0.04  (-0.33 to 0.24) | | | 0.24 | | 0.05  (-0.24 to 0.33) | 0.21 |
| Depression | | | -0.04  (-0.12 to 0.05) |  | | -0.03  (-0.11 to 0.05) | | |  | | 0.05  (-0.03 to 0.14) |  |
| Schizophrenia | | | 0.08  (-0.20 to 0.37) |  | | -0.18  (-0.47 to 0.10) | | |  | | 0.06  (-0.22 to 0.35) |  |
| Puberty stage at serum measurement | | | | | |  | | |  | |  |  |
| 1 | | | -0.01  (-0.07 to 0.04) | 0.18 | | -0.05  (-0.11 to 0.00) | | | 0.003 | | -0.03  (-0.08 to 0.03) | 0.82 |
| 2 | | | -0.01  (-0.09 to 0.08) |  | | 0.05  (-0.04 to 0.13) | | |  | | 0.04  (-0.05 to 0.13) |  |
| 3 | | | 0.07  (-0.04 to 0.17) |  | | 0.11  (0.01 to 0.21) | | |  | | 0.02  (-0.09 to 0.12) |  |
| 4-5 | | | 0.08  (-0.09 to 0.25) |  | | 0.21  (0.04 to 0.38) | | |  | | -0.08  (-0.25 to 0.09) |  |
