# Supplementary material for: Serum 25-Hydroxyvitamin D3 and D2 and Non-Clinical Psychotic Experiences in Childhood
Source: PLoS One. 2012 Jul 25;7(7):e41575. doi: 10.1371/journal.pone.0041575 (PMC3405076; doi:10.1371/journal.pone.0041575)
Supplement: Table S3 — Univariable associations between potential confounders and suspected/definite (N = 3182) and definite (N = 2974)psychotic experiences (PLIKS). (DOCX) [file pone.0041575.s003.docx]

**Supplementary Table 3.** Univariable associations between potential confounders and suspected/definite (N=3182) and definite (N=2974)psychotic experiences (PLIKS)

|  | Suspected/definite PLIKS | | | | Definite PLIKS | | | |  |  |
| --- | --- | --- | --- | --- | --- | --- | --- | --- | --- | --- |
|  | Odds ratio per SD/category change (95%CI) | | | *P* | Odds ratio per SD/category change (95%CI) | | *P* | |  |  |
| BMI (kg/m^2^) | 1.01 (0.97-1.05) | | | 0.67 | 1.00 (0.95-1.05) | | 0.98 | |  |  |
| WISC full IQ score at age 8.5 | 0.99 (0.99-1.00) | | | 0.07 | 0.99 (0.98-1.00) | | 0.049 | |  |  |
| Non-white ethnicity | 1.37 (0.49-3.82) | | | 0.55 | 1.43 (1.10-1.87) | | 0.008 | |  |  |
| Head of household social class | | | |  |  | |  | |  |  |
| i | 1.0 (reference) | | | 0.36 | 1.0 (reference) | | 0.13 | |  |  |
| ii | 1.33 (0.94-1.87) | | |  | 1.60 (0.93-2.75) | |  |  |  |  |
| iii non-manual | 0.95 (0.63-1.43) | | |  | 1.00 (0.51-1.95) | |  |  |  |  |
| iii manual | 1.58 (0.94-2.65) | | |  | 1.74 (0.78-3.87) | |  |  |  |  |
| iv/v | 1.63 (0.76-3.49) | | |  | 3.67 (1.46-9.22) | |  |  |  |  |
| Paternal education | | | |  |  | |  | |  |  |
| None/CSE | 1.0 (reference) | | | 0.006 | 1.0 (reference) | | 0.005 | |  |  |
| Vocational | 0.75 (0.43-1.29) | | |  | 1.32 (0.68-2.56) | |  |  |  |  |
| O level | 0.57 (0.38-0.87) | | |  | 0.37 (0.19-0.71) | |  |  |  |  |
| A level | 0.65 (0.45-0.94) | | |  | 0.57 (0.34-0.98) | |  |  |  |  |
| Degree | 0.55 (0.37-0.81) | | |  | 0.49 (0.28-0.87) | |  |  |  |  |
| Maternal education | | | |  |  | |  | |  |  |
| None/CSE | 1.0 (reference) | | | 0.03 | 1.0 (reference) | | 0.002 | |  |  |
| Vocational | 0.92 (0.51-1.67) | | |  | 0.74 (0.33-1.70) | |  |  |  |  |
| O level | 0.70 (0.45-1.11) | | |  | 0.65 (0.36-1.19) | |  |  |  |  |
| A level | 0.75 (0.47-1.18) | | |  | 0.48 (0.26-0.91) | |  |  |  |  |
| Degree | 0.58 (0.36-0.95) | | |  | 0.36 (0.18-0.73) | |  |  |  |  |
|  | | | |  |  | |  | | | |
| Child normally wears hat whilst out in the sun | | | |  |  | |  | | | |
| Always | 1.0 (reference) | | | 0.20 | 1.0 (reference) | | 0.38 | | | |
| Usually | 0.90 (0.64-1.26) | | |  | 0.78 (0.47-1.29) | |  |  |  |  |
| Sometimes | 0.77 (0.55-1.09) | | |  | 0.77 (0.46-1.28) | |  |  |  |  |
| Never | 0.89 (0.49-1.60) | | |  | 0.75 (0.30-1.85) | |  |  |  |  |
| Child normally wears something to cover the skin whilst out in the sun | | | |  |  | |  | | |  |
| Always | 1.0 (reference) | | | 0.05 | 1.0 (reference) | | 0.50 | | |  |
| Usually | 1.08 (0.75-1.56) | | |  | 1.04 (0.60-1.82) | |  |  |  |  |
| Sometimes/Never | 0.76 (0.52-1.12) | | |  | 0.88 (0.50-1.55) | |  |  |  |  |
| Child normally uses sunblock whilst out in the sun | | | |  |  | |  | | |  |
| Always | 1.0 (reference) | | | 0.34 | 1.0 (reference) | | 0.41 | | |  |
| Usually | 0.90 (0.69-1.18) | | |  | 0.74 (0.49-1.13) | |  |  |  |  |
| Sometimes/Never | 0.86 (0.57-1.28) | | |  | 0.93 (0.52-1.64) | |  |  |  |  |
| Child normally avoids midday sun | | | |  |  | |  | | |  |
| Always | 1.0 (reference) | | | 0.90 | 1.0 (reference) | | 0.93 | | |  |
| Usually | 0.90 (0.62-1.30) | | |  | 0.81 (0.47-1.41) | |  |  |  |  |
| Sometimes | 0.84 (0.57-1.21) | | |  | 0.97 (0.56-1.67) | |  |  |  |  |
| Never | 1.13 (0.68-1.88) | | |  | 0.87 (0.39-1.97) | |  |  |  |  |
| Time spent outdoors in summer during school weekdays >3h/day | 0.96 (0.75-1.23) | | | 0.75 | 1.17 (0.81-1.69) | | 0.412 | | |  |
| Time spent outdoors in summer during weekends >3h/day | 1.67 (1.00-2.77) | | | 0.049 | 2.36 (0.95-5.83) | | 0.063 | | |  |
| Time spent outdoors in summer during holidays >3h/day | | 1.05 (0.69-1.58) | 0.83 | | | 1.85 (0.85-4.00) | | 0.12 | | |
| Family history of depression/schizophrenia | | |  | | |  | |  | | |
| None | | 1.0 (reference) | 0.001 | | | 1.0 (reference) | | 0.001 | | |
| Depression | | 1.50 (1.16-1.94) |  | | | 1.90 (1.31-2.75) | |  | | |
| Schizophrenia | | 1.93 (0.89-4.19) |  | | | 1.83 (0.56-6.03) | |  | | |
| Puberty stage at serum measurement | | |  | | |  | |  | | |
| 1 | | 1.0 (reference) | 0.56 | | | 1.0 (reference) | | 0.62 | | |
| 2 | | 0.97 (0.73-1.30) |  | | | 0.92 (0.60-1.42) | |  | | |
| 3 | | 1.06 (0.75-1.48) |  | | | 0.99 (0.60-1.66) | |  | | |
| 4-5 | | 1.20 (0.71-2.04) |  | | | 1.42 (0.68-2.94) | |  | | |
